# ABO Incompatible Kidney Transplantation—Current Status and Uncertainties

**DOI:** 10.1155/2011/970421

**Published:** 2011-12-10

**Authors:** Milljae Shin, Sung-Joo Kim

**Affiliations:** Division of Transplant Surgery, Department of Surgery, Samsung Medical Center, Sungkyunkwan University School of Medicine, No. 50 Ilwon-dong, Kangnam-ku, Seoul 135-710, Republic of Korea

## Abstract

In the past, ABO blood group incompatibility was considered an absolute contraindication for kidney transplantation. Progress in defined desensitization practice and immunologic understanding has allowed increasingly successful ABO incompatible transplantation during recent years. This paper focused on the history, disserted outcomes, desensitization modalities and protocols, posttransplant immunologic surveillance, and antibody-mediated rejection in transplantation with an ABO incompatible kidney allograft. The mechanism underlying accommodation and antibody-mediated injury was also described.

## 1. Introduction

The most effective treatment of end-stage renal disease is kidney transplantation, but a severe donor shortage has significantly limited this treatment. To overcome this profound donor shortage, immunologic barriers historically considered as absolute contraindications to transplantation are being reevaluated. One such barrier is the ABO blood group incompatibility. Kidney transplantation across the ABO blood group barrier has the potential to expand the pool of donors, increase the availability of transplantable organs, and decrease the prolonged time on the waiting list for a kidney [1–4]. In addition, through the help of a better understanding of related immunologic mechanisms and effective various regimens for controlling it, ABO-incompatible kidney transplantation (ABOi KT) is being performed with increasing frequency [5].

To clarify the current status and uncertainties in this area, the present paper focuses on recently reported outcomes of ABOi KT, preconditioning methods before transplantation, posttransplant monitoring and management, diagnosis and treatment of antibody-mediated rejection, and the basic elucidation of immune tolerance and accommodation.

## 2. History of ABO-Incompatible Kidney Transplantation

### 2.1. Brief History of ABOi KT

The use of an ABO-incompatible (ABOi) kidney is not a recent development. The first attempt at ABOi KT was reported in 1955 by Chung et al. [6]. In their experience, eight of ten ABOi kidney allografts did not work successfully within the first few postoperative days. Although further attempts at ABOi KT have been sporadically reported, these series revealed similar poor outcomes with graft survival rates of approximately 4% at one year [7–10]. Therefore, ABOi KT was largely abandoned.

An interesting clinical trial was reported in 1987 when Thielke et al. [11] showed that 12 of 20 transplants from blood group A2 donors into O recipients maintained long-term allograft function. This procedure is based on the finding that the expression of the A antigen on the red blood cell in the A2 donor was much weaker than that in the A1 donor. Regrettably, this technique can be used only in a small minority of KT candidates.

In 1987, Alexandre et al. introduced an effective desensitization protocol to achieve success in ABOi living donor KT [11]. This protocol included pretransplant repeated plasmapheresis as a strategy not only to reduce the titers of anti-A or -B antibodies, but also to decrease the antilymphocyte globulin-based induction. This plasmapheresis also altered the triple maintenance immunosuppression of cyclosporine, azathioprine, and corticosteroids and concomitant splenectomy [12]. A one-year graft survival of 75% and a recipient survival of 88% were achieved in the 23 recipients [2]. While their results were impressive and became the basis of the next desensitization protocols for ABOi KT, the ABOi KT was still uncommon in the west.

These efforts regarding ABOi KT were significantly expanded in Japan because of the near absence of deceased donors and the only 0.15% of living A2 donors. The largest number of ABOi KT since 1989, more than 1000 cases, has been performed in Japan [13]. The percentage of ABOi KT surgeries reached 14% of all living donor KTs performed in Japan [11]. Following the remarkable results reported in the Japanese center utilizing modern desensitization techniques, together with the development of new immunosuppressive therapies, ABOi KT began receiving new interest in Europe and the USA in the early 2000s [12].

### 2.2. Published Clinical Outcomes of ABOi KT

Short-term results from the protocol described above have been notable. For instance, in the study of Tydén et al. [14], recipients with a baseline anti-A or -B IgG titer of up to 1 : 128 were successfully transplanted with no episode of acute rejection. Montgomery [15] reported one-year patient and graft survivals of 96.3% and 98.3%, respectively, in a cohort of 60 consecutive ABOi KTs using a variety of protocols. Oettl et al. [16] demonstrated a 100% survival rate of both patients and grafts at one-year after transplant.

Moreover, long-term results of ABOi KT reported by western and Japanese transplant centers also have shown that ABOi KT is equivalent to ABO-compatible KT [12]. Genberg et al. [17] reported that ABOi KT had no negative impact on long-term graft function compared to that of ABO-compatible KT in terms of patient survival, graft survival, or incidence of acute rejection after a mean followup of three years. Tydén et al. [18] found that graft survival was 97% for the ABOi KT compared with 95% for the ABO-compatible KT in their three-center experience at their five-year followup. Patient survivals were 98% in both KT groups.

In the analyzed UNOS data of Gloor and Stegall [19], they concluded that a long-term immunological response against ABO incompatibility has little effect on graft survival with current immunosuppressive protocols and patient monitoring. Tanabe [20] summarized the outcomes of 851 ABOi KT performed in 82 institutions in Japan between 1989 and 2005. The five-year graft survival in their study was 79%, with patient survival at 90%. Montgomery [15] obtained five-year patient and graft survivals of 89% following ABOi KT at Johns Hopkins Hospital. Fuchinoue et al. [21] report the five-year outcome of ABOi KT as a graft survival rate of 100%, whereas Ishida et al. [13] achieved a graft survival of 57% and patient survival of 89% at ten years postoperatively for more than 130 cases of ABOi KT.

## 3. Current Status of ABO-Incompatible Kidney Transplantation

### 3.1. General Methodology of Desensitization

For the successful performance of ABOi KT, the antibody-mediated response must be understood and targeted. Over the past 20 years, several strategies have been developed to resolve and modulate this response. These strategies, or desensitizations, are all based on the same principles [5, 12, 22], including not only the removal of preexisting antibodies that are directed at the donor ABO antigen, but also waiting to transplant until the anti-ABO antibody titer is below a set target. Additionally, the prevention of further production of new recipient anti-ABO antibodies before and after transplantation is another founding principle. 

Ordinarily, several pretransplant apheresis sessions are required for antibody removal. To prevent reformation of the antibody, apheresis is followed by intravenous immunoglobulin, a mixture of immunosuppressive therapies, and erasable splenectomy [11, 12]. This procedure usually occurs over a period of one to two weeks.

### 3.2. Specific Preconditioning Modality

#### 3.2.1. Antibody Depletion Technique

In the field of ABOi KT, currently used antibody depletion techniques include therapeutic plasma exchange, double-filtration plasmapheresis, and antigen-specific immunoadsorption. The great difference among these techniques is their degree of selectivity [12].

The simplest and most common method to remove antibody from plasma is therapeutic plasma exchange, in which large amounts of plasma are withdrawn and replaced with colloid solutions [23]. This procedure eliminates approximately 20% of the anti-ABO antibodies with each session. However, this technique is not sufficiently selective to remove only protective antibodies and also removes coagulation factors, hormones, and antiviral and antibacterial immunoglobulin G (IgG) and immunoglobulin M (IgM). The removal of these factors increases the risk of bleeding or infection [24]. However, this technique is by far the least expensive means of removing antibodies [12].

The selective techniques of double-filtration plasmapheresis or antigen-specific immunoadsorption are safe and more effective and are therefore usually the first choice. Because no coagulation factors are eliminated, large plasma volumes can be processed, and the resultant efficacy is increased compared to that of therapeutic plasma exchange [12]. Using a second filter, double-filtration plasmapheresis is capable of eliminating the plasma fraction containing the immunoglobulins [12] and decreases the amount of plasma discarded [23]. Using the process of immunoadsorption, the plasma is processed through a Glycosorb ABO immunoadsorbent column and reinfused into the patient. There are no volume losses, and thus the number of adsorption cycles has no limit [23]. Although the ABOi KT is an American Society for Apheresis (ASFA) category II indication, there has been no clinical trial differentiating antibody reduction procedures or the standardized monitoring protocol [4]. 

#### 3.2.2. Intravenous Immunoglobulin

Intravenous immunoglobulin plays a role in the downregulation of the antibody-mediated immune response [5]. The immunoglobulin blocks not only the Fc receptor on the mononuclear phagocyte, but also the direct neutralization of the alloantibody. Further, it inhibits the CD19 expression on the activated B cell, as well as that of the complement and the alloreactive T cell. Although alloantibody rebounds within days of the discontinuation of plasmapheresis, the benefit of intravenous immunoglobulin may continue for many months after drug administration.

#### 3.2.3. Splenectomy

Traditionally, concurrent splenectomy was an important prerequisite of the desensitization protocol for ABOi KT, based on the idea that it contributed to the reduction of the antibody-producing B-cell pool [5]. Alexandre et al., the early investigator of ABOi KT, suggested that the splenectomized recipient had a much smaller risk of antibody-mediated rejection. 

However, whether splenectomy is essential for successful ABOi KT remains unproven. Sonnenday et al. [2] found that the suppression of anti-ABO antibody after splenectomy was not significantly different from that of nonsplenectomized patients. Sonnenday et al. [2] reported that splenectomized recipients had a 25% greater mortality at 84 months compared with that of non-splenectomized recipients. Gloor et al. [25] reported that splenectomy is not necessary even for patients with high-baseline anti-ABO antibody titers. Takahashi et al. [26] demonstrated that splenectomy is not necessary to inhibit antibody production because significant numbers of memory cells exist in the bone marrow.

There is a growing consensus that splenectomy is not necessary in ABOi KT recipients [19]. Most investigations have commonly substituted anti-CD20 monoclonal antibody induction [5].

#### 3.2.4. Anti-CD20 Monoclonal Antibody

The anti-CD20 monoclonal antibody, rituximab, directly inhibits B-cell proliferation and induces cellular apoptosis through the binding of complement. Complement, in turn, mediates antibody-dependent cell-mediated cytotoxicity and subsequent cell death [27]. 

Several centers have established the use of rituximab as a chemical splenectomy due to its potent ablation of the B-cell compartment [20]. The advantage of rituximab over splenectomy is that it ablates the B cell during the period of the greatest risk of antibody-mediated rejection and then allows the humoral immune system to heal with an intact spleen [28]. Fuchinoue et al. [21] reported that patients who received rituximab induction had a lower incidence of acute antibody-mediated rejection and better outcome of five-year graft survival compared to those in ABO-compatible or -incompatible KT recipients who were not treated with rituximab.

However, rituximab's mechanism of action in ABOi KT is not clear. Since CD20 antigen is not expressed on plasma cells, rituximab is effective against pre-B and B-cells but not against plasma cells, which contributes to antibody production [26]. Some data [24] suggest that rituximab has a much weaker impact on the memory B cell population, the progenitor of the IgG-secreting plasma cell. The appropriate dosage and the initial time for administration were also unclear. Toki et al. [29] indicated that a low-dose rituximab less than 375 mg/m^2^ has a potent impact on the depletion of B cells in the spleen and peripheral blood. Toki et al. [29] demonstrated that a single dose of rituximab, even at 50 mg/m^2^, depleted B cells from the peripheral blood as effectively as did the 375 mg/m^2^ dose. Fuchinoue et al. [21] revealed that there was no difference in serum creatinine at one year after transplant, irrespective of the dose of rituximab.

As an extension of this idea, these have led to a modified protocol that consists of an antibody-depleting procedure and intravenous immunoglobulin with no long-term B-cell suppression from splenectomy or rituximab. Flint et al. [30] and Montgomery et al. [31] have applied this protocol to their cohort studies. Montgomery's results showed that the five-year graft survival rate was 88.7% with no instance of antibody-mediated rejection or graft loss.

#### 3.2.5. Quadruple Immunosuppression

Immunosuppressive regimens are required for both T-cell-mediated immunity and B-cell-mediated immunity, which are similar to that used for ABO-compatible KT [26]. Calcineurin inhibitors (cyclosporine and tacrolimus) and antimetabolites (mycophenolate mofetil and azathioprine) are mainly used with low-dose steroids. In addition, monoclonal or polyclonal antibody agents (anti-CD25 antibody or antithymocyte globulin) are also often used during the induction period. Tanabe [20] started to use tacrolimus in combination with mycophenolate mofetil as a basic immunosuppressant after ABOi KT and reported a greatly improved graft survival compared with that of cyclosporine administration.

Antimetabolites seem to take seven~ten days to be efficient as *in vivo* immunosuppressants. Therefore, immunosuppressive therapy as desensitization should be started before transplantation in order to adequately inhibit antibody production [20].

### 3.3. Current Desensitization Protocols

In order to achieve a successful outcome with ABOi KT, many centers have employed their own independent desensitization protocols. Although there are slight differences in the preconditioning formalities depending on the transplant center, most include a combination of pretransplant plasmapheresis, intravenous immunoglobulin, and tacrolimus-mycophenolate-based immunosuppression with antibody induction. Splenectomy or rituximab administration is used selectively. After transplant, very close monitoring of the anti-ABO antibody titer is typically carried out for a minimum of two weeks. If necessary, plasmapheresis is added to eliminate the rebounding antibody level [5, 15]. The details are shown in Table 1.

## 4. Uncertainties in ABO-Incompatible Kidney Transplantation 

### 4.1. Graft Accommodation and B-Cell Tolerance

If anti-ABO antibodies are removed prior to transplantation, one of three types of immune response may occur: rejection, immune tolerance, or accommodation [32]. (1) About 2–5% of patients produce antibodies to the incompatible ABO antigen, which mediate allograft rejection. (2) Some recipients seem to have immunologic tolerance to the incompatible ABO antigen because they do not reject the allograft or produce anti-ABO antibody against it. (3) The others display an accommodation state regarding the allograft [33].

Theoretically, natural anti-ABO antibody might be inducing antibody-mediated rejection in ABOi KT [26] and can manifest as a hyperacute rejection or as an acute or delayed humoral rejection. Antibody-mediated damage can result in rapid and irreversible graft thrombosis due to complement activation or contributes to long-term graft dysfunction [34]. However, as the anti-ABO antibody usually reaccumulates and persists after successful ABOi KT, the recipient maintains satisfactory graft function. This resistance of allograft to antibody-mediated rejection despite the significant presence of anti-ABO antibodies in the recipient serum is known as accommodation [1, 5, 35, 36]. 

Park et al. [1] defined the criteria of accommodation in ABOi KT to include (1) detectable anti-ABO antibody in the recipient serum, (2) normal graft histology according to light microscopy, (3) the presence of A or B antigen in the graft, and (4) renal function similar to that of ABO compatible patients (GFR greater than 45 mL/min at one-year after transplant). In 2006, the American Society of Transplantation reached a consensus on accommodation, stating that it occurred when C4d deposition was detected with normal function and structure of the graft [35].

How accommodation is induced and through what mechanism it is maintained are not well understood. Various hypotheses have been proposed to describe the mechanism responsible for accommodation [34, 37], including changes in the function of antidonor antibody, changes in the antigen, acquired resistance of the allograft through the expression of an antiapoptotic gene, or an expression of complement regulatory protein.

The study of Ishida et al. [33] presented the difference in quality between antibodies produced by accommodated and non-accommodated recipients. Kirk et al. [38] suggested that accommodation is related to a shift from the IgG isotype to the IgG2 isotype that is less effective at activating complement and that competitively inhibits the binding of the more cytotoxic isotype. Chang and Platt [39] discovered that healthy organs could absorb antidonor antibody in large amounts, for which the accommodated functioning graft served as a sink. According to these results, accommodation may reflect a change in the properties of the antibody or antigen. 

Park et al. [1] and Delikouras and Dorling [40] reported that the Bcl-2 and Bcl-xl, antiapoptotic molecules, were found in the accommodated ABO incompatible kidney graft. However, Bax, a proapoptotic marker, was not detected. Salama et al. [41] demonstrated upregulation of Bcl-xL in the microvascular endothelial cells of accommodated grafts. These findings are consistent with the hypothesis that the endothelium of the kidney allograft will be initially exposed to low titers of anti-ABO antibody, which will in turn instigate a series of protective changes that manifest as accommodation. 

Consistent with this concept, data from Stegall et al. [42] suggest that a decrease in tumor necrosis factor-*α* and alteration in SMAD (mothers against decapentaplegic homolog) gene expression may be important in long-term accommodated grafts. Griesemer et al. [35] introduced the concept that the upregulation of a complement regulatory protein such as CD59 seems to be involved in accommodation by preventing the formation of the membrane attack complex (MAC) on the accommodated graft.

Accommodation is different from immune tolerance. The accommodated allograft kidney remains protected even though it is transplanted into a new recipient. However, an immunotolerant allograft preserves the potential to reject the tissue from the same donor. Immune tolerance is a state of immune unresponsiveness to the presence of specific non-self-antigens in the absence of long-term immunosuppression. Published studies have provided evidence that B cells have an important role in tolerance. Kirk et al. [38] postulated that the prolonged depletion of alloreactive B cells in tolerant mice is achieved through the dominant and active suppression of T helper cells. Ogawa et al. [32] suggested that prolonged T-cell suppression in the ABOi KT recipient may result in a similar induction of tolerance to that of the incompatible ABO antigen.

### 4.2. Posttransplant Monitoring and Desensitization (Posttransplant Immunologic Surveillance)

The monitoring of anti-ABO antibody titer is critical for determining the effectiveness of desensitization and the optimum time to permit graft implantation. After transplantation, the anti-ABO antibody level must be monitored to detect its reaccumulation, which may indicate or induce antibody-mediated rejection. In patients with a higher rebound in serum antibody production after the incompatible transplant, desensitization therapy, especially antibody-depletion procedures, should be repeated. Most studies [3, 43] agree that posttransplant DFPP was ineffective at preventing the rebound of anti-ABO antibody titers compared to the efficacy of therapeutic plasma exchange. 

The Jones Hopkins group of Montgomery et al. [15, 28] determined the initiation of posttransplant plasmapheresis based on the pretransplant baseline antibody level before preconditioning. Others [44] have granted that preemptive posttransplant plasmapheresis may be dispensable, favoring an on-demand strategy according to the post-transplant antibody titer elevation. However, there are conflicting opinions on which antibody titer is meaningful and how long antibody monitoring should continue.

Kayler et al. [45] found that all patients whose posttransplant antibody titer remained below 1 : 8 exhibited stable renal function. Those patients who had an increased titer above 1 : 64 experienced allograft failure. Stegall et al. [42] recommended initiating plasmapheresis if the antibody titer increases to 1 : 16 in the first two weeks after transplantation. Gloor et al. [25] showed that humoral rejection was rare when the antibody level was maintained less than 1 : 8 in the first week and 1 : 16 in the second week after transplantation. They then allowed antibody titers to rise if the graft function and surveillance biopsies were normal. Takahashi [46, 47] asserted that anti-ABO antibody titers should be suppressed to the lowest level during the first week after transplant, when surface antigenicity is increasing in the vascular endothelial cells of the graft kidney.

On the other hand, Park et al. [1] demonstrated that anti-ABO antibody titers return to detectable levels in all accommodated and nonaccommodated recipients, even in the absence of humoral rejection or chronic graft damage. Tobian et al. [3] also demonstrated that the clinical significance of an increased posttransplant anti-ABO antibody level is variable, and that there was no dependable correlation for antibody-mediated rejection. These findings are supported by the fact that a high titer of antibody is generally necessary but is not sufficient for antibody-mediated rejection.

### 4.3. Acceptable Anti-Blood Group Titer at the Time of Transplant

Before the initiation of preconditioning, the baseline anti-ABO antibody titer is well known as a significant predictor of the severity of antibody-mediated graft injury as well as graft survival [19, 42]. Although a few recent reports have shown that a high-baseline antibody titer is no longer predictive for poor graft outcome in patients that received tacrolimus- or mycophenolate mofetil-based immunosuppression [6, 19], antibody removal should be as complete as possible. 

Most centers performing ABOi KT have adhered to the guideline that serum anti-ABO antibody titers should be 1 : 16 or lower before transplantation [26]. However, the acceptable upper limit of anti-ABO antibody titers is exclusively based on empirical evidence, not substantiated by deductive reasoning. Wilpert et al. [48] decreased the antibody titer to below 1 : 4 before transplant, while Chung et al. [6] chose a limit of 1 : 32 in their preconditioning protocol.

Some institutions use the antiglobulin IgG antibody titer endpoint as the critical titer when assessing patients before and after transplantation. Others consider both IgM and IgG antibody titers [4]. Which type of anti-ABO antibody, IgM or IgG, causes antibody-mediated graft damage due to the unique characteristics of the antibody remains unclear [49]. If anti-ABO IgG antibodies are believed to be responsible for worse graft outcomes, it is expected that blood group O recipients will be more likely to suffer graft damage than will A or B recipients.

### 4.4. Antibody-Mediated Rejection

Antibody-mediated rejection (ABMR) is known as the primary cause of graft loss in ABOi KT. It is clear that ABMR has a negative influence on short-term outcome following ABOi KT. Recent studies have reported that ABMR occurred in 17.9% up to 30% of ABO-incompatible kidney transplants [22, 50, 51]. Toki et al. [50] demonstrated that anti-ABO IgG antibody titers of 1 : 32 at the time of transplantation and the presence of donor-specific anti-HLA antibodies were independent risk factors for ABMR. Although the development of desensitization protocols has improved graft survival [50], the outstanding results are largely due to aggressive surveillance, early detection, and an enhanced therapeutic approach for ABMR [45]. 

The hyperacute rejection due to anti-ABO antibody does not occur within 24 hours, which is called the silent period. The greatest incidence of acute antibody-mediated rejection occurs two to seven days after transplant and does not typically occur after more than one month. Some researchers, therefore, have defined the first two weeks after transplantation as the critical period during which accommodation is usually induced and established. Once accommodation is established, acute antibody-mediated rejection does not occur, leading to the stable period [26]. 

Takahashi [26] classified acute ABMR into two types on the basis of antigen stimulation. Type I ABMR is caused by resensitization due to recovery of the ABO antigen. ABO antigen in the graft directly stimulates immunological responses, resulting in an explosive antibody production early in the critical period, typically, IgG antibody. Type II ABMR is caused by primary sensitization due to an ABO blood-group-associated antigen. In response to bacterial infection, an ABO-antigen-like substance is found on the surfaces of bacterial cells, acting as a cross-reacting antigen to cause sensitization and antibody production, mainly IgM. This type II rejection usually progresses more slowly and is less severe than is type I rejection.

### 4.5. Diagnosis of Acute Antibody-Mediated Rejection

Clinically, ABMR was suspected when the serum creatinine level was increased relative to the previous value, together with a decrease in urine output. Renal biopsy is typically performed in such suspected cases [21]. Acute ABMR after ABOi KT is diagnosed on the basis of morphologic, immunohistologic, and serologic features. Morphologic evidence includes (1) leukocyte (neutrophil, monocyte, and macrophage) infiltration into the peritubular capillary and/or glomeruli; (2) arterial fibrinoid necrosis; (3) glomerular and arterial thrombi; (4) acute tubular injury. Immunohistologic evidence involves (1) peritubular capillary C4d deposition and (2) immunoglobulin and/or complement in arterial fibrinoid necrosis. For serologic evidence, circulating specific antidonor antibodies at the time of biopsy should be found. Overall, at least one finding in each of the three categories must be present for a biopsy to be diagnosed as acute ABMR [22, 25, 52]. These diagnostic criteria were established by the National Institutes of Health and the Banff working group. The former group also requires clinical evidence of graft dysfunction, while the latter group accepts the possibility of subclinical acute ABMR, defined as C4d staining and leukocyte margination in the peritubular capillary in a protocol biopsy of a well-functioning graft [52].

Antibody-mediated rejection is thought to be caused by endothelial cell activation in the graft [39]. Peritubular capillary C4d deposition has been considered to be an important histologic indicator of antibody-endothelial cell interaction and is a key element in the diagnosis of ABMR. The presence of donor-specific antibody or the presence of C4d alone in the peritubular capillary is not diagnostic of acute ABMR in the setting of ABOi KT [5, 19]. Racusen and Haas [52] reported that C4d staining was associated with ABMR and graft injury in the malfunctioning graft, whereas it reflected graft accommodation in the stably functioning graft. Setoguchi et al. [53] detected C4d staining in the peritubular capillary in 94% of their protocol biopsy specimens but in only 28% of subclinical ABMR cases. Haas et al. [54, 55] observed C4d deposition in 80% of protocol biopsies in the absence of allograft dysfunction or other histologic abnormalities suggestive of acute ABMR. Meanwhile, they [54] suggested that deposition of C3d, alone or in combination with C4d, may identify a more severe form of ABMR associated with a high risk of graft loss.

### 4.6. Treatment Strategy for Acute Antibody-Mediated Rejection

Basically, ABMR was treated through the reinitiation of plasmapheresis to remove circulating antibodies [19, 53]. Plasma exchange for the treatment of rejection was first reported in 1977 [43]. Just et al. [56] reported an immunoadsorption-based protocol to eliminate deposited IgG from the allograft.

Standard treatment for ABMR consists of repeated plasmapheresis-plasma exchange or immunoadsorption and intravenous immunoglobulin [57]. Various combinations of these therapeutic modalities have been successfully used to treat ABMR and improve outcomes. Most institutes [25, 58, 59] treated ABMR with a series of plasma exchanges followed by low-dose IVIG in addition to methylprednisolone until clinical improvement was achieved or until ABMR was histologically determined to have been resolved. Racusen and Haas [52] reported that reversal rates for ABMR were approximately 90% using such protocols, contrasted to reversal rates of less than 50% with traditional immunosuppression alone. Rituximab, an immunosuppressive agent which controls antidonor antibody production, and several complement inhibitors have also been reported to obtain significant efficacy [60].

The use of rituximab is intended to deplete B cells and thereby suppress antibody production. Several doses of rituximab at 375 mg/m^2^ were intravenously administered to resolve ABMR [27]. Garrett et al. administrated rituximab as a first-line therapy for ABMR. Sarwal et al. noted that allografts with CD20^+^ cells in biopsy specimen were strongly associated with the clinical phenotype of glucocorticoid resistance and chose to treat ABMR with rituximab.

Together with tacrolimus-mycophenolate rescue, the majority of AMBR cases have received antithymocyte globulin at the time of plasmapheresis [57]. Toki et al. [50] administered OKT3 at a dose of 5 mg/day for seven days in patients with persistent antibody-mediated rejection.

Previously mentioned anti-ABMR therapies including plasmapheresis, intravenous immunoglobulin, antithymocyte globulin, and rituximab have provided suboptimal results [61]. One reason for this insufficient success is that they do not exert direct effects on the mature plasma cell. A proteasome inhibitor, such as bortezomib, depletes both transformed and nontransformed plasma cells in animal and human transplant recipients. Recent reports have also shown that eculizumab, a humanized monoclonal antibody against terminal complement protein C5, is an effective therapy to inhibit terminal complement activation and prevent antibody-induced injury. All of these modes are useful in the treatment of refractory ABMR [24, 57].

Splenectomy may be a possible option as a rescue treatment for severe ABMR resistance to standard treatment after ABOi KT. Kaplan et al. [60] reported the first early experience with rescue splenectomy and suggested that the procedure may specifically and irreversibly deplete memory B cells, thus offering an additive effect to the standard treatment.

### 4.7. Chronic Antibody-Mediated Rejection

Although successful strategies have been developed to treat acute ABMR, humoral alloreactivity in the early posttransplant period adversely impacts long-term allograft survival and contributes to chronic rejection [54]. Toki et al. [50] revealed that a high panel-reactive antibody value was significantly associated with the development of chronic ABMR characterized by transplant glomerulopathy. Recent studies [19, 50] have found that a prior history of ABMR was significantly associated with the development of transplant glomerulopathy, with an incidence of 22% at one year after transplantation. Smith et al. [37] clarified sequential stages of chronic ABMR using an animal model and determined that the first symptom was circulating alloantibody, C4d, or a combination of the two. Only rarely was transplant glomerulopathy observed in the absence of C4d or alloantibody.

The National Institutes of Health suggested diagnostic criteria for chronic ABMR in ABOi kidney allograft [52]. Their criteria require three of the following four lesions: (1) arterial intimal fibrosis, (2) interstitial fibrosis/tubular atrophy, (3) duplication of the glomerular basement membrane, or (4) lamination of the peritubular capillary basement membranes.

### 4.8. Minimizing Immunosuppressive Therapy

ABOi KT is considered an increased immunologic risk; therefore, aggressive immunosuppressive protocols traditionally have been used [11]. Most centers have adopted polyclonal antibody for the induction and chronic maintenance of immunosuppression based on tacrolimus and mycophenolate, starting two weeks before the transplantation.

Nevertheless, Chuang et al. [62] showed that maintenance-immunosuppressive therapy did not affect isoagglutinin titer in ABOi KT. In addition, no significant difference in isoagglutinin titer was observed between tacrolimus and cyclosporine groups. Flint et al. [30] demonstrated that only standard immunosuppression could produce a successful ABOi KT as long as an adequate desensitization protocol was employed. Far from high immunologic risk, avoidance of excessive immunosuppression potential is a benefit to ABOi KT recipients. Magee [5] avoided lymphocyte-depleting antibody because it is no more effective in preventing ABMR and is adversely associated with a higher incidence of infective complications.

The long-term need for steroids remains a question in ABOi KT. Crew and Ratner [24] and Galliford et al. [58] reported that ABOi KT can be successfully accomplished using a steroid-sparing regimen without resulting steroid-resistant rejection.

## 5. Conclusions

The idea that ABO blood group incompatibility should be considered an absolute contraindication to kidney transplantation has been challenged in the past two decades. As the pretransplant and posttransplant desensitization protocols have developed and changed in many different fields, satisfactory results have been observed. As the body of immunologic knowledge including that regarding antibody-mediated rejection has grown, the allograft outcomes have been enhanced. Overall success rates are now comparable with those of ABO-compatible kidney transplantation. Due to the surprising result, the pool of potential ABOi KT candidates has increased.

 Because the long-term outcome of ABOi KT has not yet been determined, uncertain and contentious ideas regarding it use still exist. Despite this, ABOi KT has become one of the established therapies. These inspiring circumstances forecast a hopeful future for ABO-incompatible kidney transplantation.

## Figures and Tables

**Table 1:** Several examples of current desensitization protocols.

	Pretransplant desensitization				Acceptable final titer	Posttransplant desensitization				Posttrans- plant monitoring	Splenectomy
	Ab depletion	IVIG	Rituximab	IS drug		Ab depletion	IVIG	Rituximab	IS drug		
Montgomery, 1st era (Johns Hopkins)	PP or IA	Low dose* (0.1 g/kg): CMV-IVIG	No: if high risk, single dose at POD# −1	FK/MMF: start at the beginning of PP	<1 : 16	PP or IA	Low dose (0.1 g/kg): CMV-IVIG	No	Daclizumab: initial 2 mg/kg, and then 1 mg/kr q 2 wks for 5-dose FK/MMF/MPD	Anti-ABO IgG titer: weekly for POD# 1 mon at POD# 2, 3, 6, 12 mon	No (work as rescue therapy for ABMR)

Tyden (Stockholm)	IA: at POD# −6, −5, −4, −1	Standard dose (0.5 g/kg): single dose at POD# −1	Yes (375 mg/m^2^): single dose at POD# −10	FK/MMF/MPD (high dosage): start at POD# −10	<1 : 8	IA: preemptive 3 times at each 3 days	No	No	FK/MMF/MPD	Anti-ABO IgG titer	No

Genberg (Stockholm)	IA	Standard dose (0.5 g/kg): single dose at POD# −1	Yes (375 mg/m^2^): single dose at POD# −30	FK/MMF/MPD (high dosage): start at POD# −10	no	IA: preemptive 3 times	Low dose (0.5 g/kg): 5 doses	No: If high B cell count, add dose	FK/MMF/MPD	B-cell count measurement at posttransplant 6 month	No

Wilpert (Germany)	IA	Standard dose (0.5 g/kg): single dose at POD# −5 ~ −1	Yes (375 mg/m^2^): single dose at POD# −30	FK/MMF/MPD: start at POD# −7	≤1 : 4	IA	No	No	basiliximab FK/MMF/MPD	Anti-ABO IgG titer: ≥1 : 8 in 1st week and ≥1 : 16 in 2nd week	No

Flint (Australia)	TPE	Low dose* (0.1 g/kg): but, 0.5 g/kg at POD# −1	No	MMF: start at POD# −10	≤1 : 8	TPE	Low dose (0.1 g/kg)	No	basiliximab FK/MMF/MPD	Anti-ABO IgG titer: daily for the first 2 weeks and then, twice a week for the first 2 months	No (work as rescue therapy for ABMR)

Gloor (Mayo)	TPE	Low dose* (0.1 g/kg)	Yes (375 mg/m^2^): 2 doses at the starting of PP	MMF	≤1 : 8	No	No	No	ATGAM FK/MMF/MPD		No

Oettl (Basel)	IA (daily)	Standard dose (0.5 g/kg): single doseat POD# −1	Yes (375 mg/m^2^): single dose at POD# −30	FK/MMF/MPD: start at POD# −14	≤1 : 8 (IgM, IgG)	IA	No	No	basiliximab FK/MMF/MPD	Anti-AB IgM or IgG titer ≥1 : 8	No

Tanabe (Tokyo)	DFPP: start at POD# −7	No	Yes (200 mg/m^2^): single dose	FK/MMF/MPD: start at POD# −7	≤1 : 32	No	No	No	basiliximab FK/MMF/MPD	No	Yes (selectively)

Montgomery, 2nd era (Johns-Hpokins)	PP	Low dose* (0.1 g/kg): CMV-IVIG	No	FK/MMF: start at the beginning of PP	≤1 : 16	PP: preemptive 2 times	Low dose (0.1 g/kg): CMV-IVIG	No	daclizumab FK/MMF/MPD	Anti-ABO IgG titer >1 : 32 (If so, protocol biopsy)	No

Galliford (UK)	PP: start at POD# −14	Low dose* (0.1 g/kg)	Yes (1 g): 2 dose at the starting of PP	FK/MMF: start at the beginning of PP	≤1 : 4	PP: at POD# 1, 3	Low dose (0.1 g/kg): 2 times routinely	Yes (1 g): at POD# 0 (posttra- nsplant)	daclizumab FK/MMF/MPD (steroid-sparing protocol)	Anti-ABO IgG titer	No

Uchida (Osaka)	DFPP or TPE	No	Yes (150 mg/m^2^): 2 dose at POD# −14, −1	FK: start at POD# −3 MMF/MPD: start at POD# −30	≤1 : 16	No	No	No	basiliximab FK/MMF/MPD	No	Yes

*IVIG after antibody depletion (a set of PP/IVIG as baseline titer) (PP: plasmapheresis, IA: immunoadsorption, TPE: therapeutic plasma exchange, DFPP: double-filtration plasmapheresis, IVIG: intravenous immunoglobulin, CMV-IVIG; CMV hyperimmune IVIG, ABMR: antibody mediated rejection, IS: immunosuppression, FK: tacrolimus, MMF: mycophenolate mofetil, MPD: methylprednisolone).
